# Investigating the Distribution Dynamics of the *Camellia* Subgenus *Camellia* in China and Providing Insights into *Camellia* Resources Management Under Future Climate Change

**DOI:** 10.3390/plants14071137

**Published:** 2025-04-06

**Authors:** Yue Xu, Bing-Qian Guan, Ran Chen, Rong Yi, Xiao-Long Jiang, Kai-Qing Xie

**Affiliations:** 1 College of Forestry, Central South University of Forestry and Technology, Changsha 410004, China; xyue03653@gmail.com (Y.X.); guanbqq@163.com (B.-Q.G.); chenran329@163.com (R.C.); 2 College of Agriculture and Biological Sciences, Dali University, Dali 671003, China

**Keywords:** *Camellia*, ensemble model, climate change, ornamental species

## Abstract

Rapid climate change has significantly impacted species distribution patterns, necessitating a comprehensive understanding of dominant tree dynamics for effective forest resource management and utilization. The *Camellia* subgenus *Camellia*, a widely distributed taxon in subtropical China, represents an ecologically and economically important group of woody plants valued for both oil production and ornamental purposes. In this study, we employed the BIOMOD2 ensemble modeling framework to investigate the spatial distribution patterns and range dynamics of the subgenus *Camellia* under projected climate change scenarios. Our analysis incorporated 1455 georeferenced occurrence records from 15 species, following the filtering of duplicate points, along with seven bioclimatic variables selected after highly correlated factors were eliminated. The ensemble model, which integrates six single species distribution models, demonstrated robust predictive performance, with mean true skil l statistic (TSS) and area under curve (AUC) values exceeding 0.8. Our results identified precipitation of the coldest quarter (Bio19) and temperature seasonality (Bio4) as the primary determinants influencing species distribution patterns. The center of species richness for the subgenus *Camellia* was located in the Nanling Mountains and eastern Guangxi Zhuang Autonomous Region. The projections indicate an overall expansion of suitable habitats for the subgenus under future climate conditions, with notable scenario-dependent variations: distribution hotspots are predicted to increase by 8.86% under the SSP126 scenario but experience a 2.53% reduction under the SSP585 scenario. Furthermore, a westward shift in the distribution centroid is anticipated. To ensure long-term conservation of *Camellia* genetic resources, we recommend establishing a germplasm conservation center in the Nanling Mountains region, which represents a critical biodiversity hotspot for this taxon.

## 1. Introduction

Biodiversity loss caused by climate change and human activities is one of the most pressing global challenges facing humanity today [1]. Climate warming, increased drought frequency, extreme weather events, and expanding desertification have significantly altered species distributions [2,3]. For example, a comparison of plant cover surveys (1977 vs. 2006–2007) along a 2314 m elevation gradient in Southern California’s Santa Rosa Mountains revealed that dominant plant species shifted upward by approximately 65 m [4]. This elevational shift coincided with regional climate changes recorded during the 30 years preceding the later survey, including warming temperatures, increased precipitation variability, and reduced snowfall, demonstrating climate change’s substantial impact on species distribution. According to the Intergovernmental Science-Policy Platform on Biodiversity and Ecosystem Services (IPBES), approximately 25% of organisms are currently threatened with extinction [5]. Understanding species distribution dynamics is essential for developing effective conservation strategies and sustainably utilizing species resources. This is particularly critical for keystone forest tree species, as their population dynamics can trigger cascading effects throughout ecosystems due to their central ecological roles [6]. Moreover, native plants, in stark contrast to plantations with simple structures, are far more effective in supporting biodiversity conservation and are unparalleled in realizing a wide range of crucial ecosystem services [7,8]. These services include significant surface carbon storage, highly efficient soil conservation, and excellent water conservation [8].

Species distribution modeling (SDM), also referred to as ecological niche modeling, integrates species occurrence data with environmental variables via statistical and machine learning algorithms to predict potential species distributions [9,10]. This approach has been widely applied in biogeography and conservation biology to assess species distribution shifts under climate change scenarios, identify biodiversity hotspots, and determine suitable areas for ex situ conservation of threatened species [9,11,12,13]. Among the various SDM approaches, the Maximum Entropy Model (MAXENT) has gained widespread popularity due to its user-friendly interface and high predictive accuracy. Other commonly used models include Generalized Additive Models (GAMs), Artificial Neural Networks (ANNs), Surface Range Envelopes (SREs), Flexible Discriminant Analysis (FDA), and Random Forests (RFs). However, the performance of these models varies depending on their underlying algorithms and assumptions [14]. To address model uncertainty and enhance predictive accuracy, the R package BIOMOD2 has been developed to integrate multiple modeling approaches, generating ensemble predictions through consensus methods such as boundary value analysis, commonality assessment, or probability weighting [15,16,17].

*Camellia* (Theaceae), a genus comprising approximately 280 species, is primarily distributed across the tropical and subtropical regions of central, southern, and southwestern China. It represents a key component of China’s subtropical evergreen broad-leaved forests (EBLFs) and serves as a valuable source of woody oilseeds and ornamental trees [18]. The genus *Camellia* is taxonomically divided into four subgenera based on morphological characteristics, including tegument differentiation and ovary compartment number: *Protocamellia*, *Camellia*, *Thea*, and *Metacamellia* [19]. The subgenus *Camellia* is further classified into seven sections: Sect. *Camellia*, Sect. *Furfuracea*, Sect. *Luteoflora*, Sect. *Oleifera*, Sect. *Paracamellia*, Sect. *Pseudocamellia*, and Sect. *Tuberculata* [20]. Many species within the subgenus *Camellia* are economically important and are being utilized for beverages, ornamental purposes, and oilseed production. For example, the seeds of *Camellia oleifera* and *Camellia vietnamensis* are rich in oil, making them valuable raw materials for edible oils, industrial oils, and cosmetic products [21]. Species within Sect. *Camellia*, such as *Camellia japonica*, *Camellia pitardii*, and *Camellia reticulata*, are widely cultivated for their striking floral beauty and vibrant colors, making them highly prized ornamentals [22]. Additionally, Sect. *Furfuracea* has significant potential for landscape applications [23]. The diverse ecological and economic applications of the subgenus *Camellia* underscore its dual significance as both an ecologically vital component of EBLFs and a valuable resource for human livelihoods.

Recent studies suggest that ongoing global climate change will significantly influence the distribution patterns of subgenus *Camellia* species. For example, research on *C. edithae* predicts a contraction in its distribution range under future climate scenarios with increasing carbon emissions [24]. In contrast, *C. reticulata* is projected to benefit from climate warming, with its suitable distribution area expected to expand northward [25]. Current research primarily focuses on the distribution dynamics of individual species within the subgenus *Camellia*. However, the species diversity distribution patterns across the entire subgenus and their distribution dynamics under future climate change remain unclear.

This study combines extensive distribution data of *Camellia* subgenus species with BIOMOD2 ensemble modeling to achieve three objectives: (1) identify key climatic drivers governing the subgenus’ distribution patterns; (2) map current biodiversity hotspots of Chinese *Camellia*; and (3) project future distribution shifts under climate change scenarios. As a keystone lineage in China’s subtropical evergreen broad-leaved forests (EBLFs), *Camellia*’s distribution dynamics hold critical implications. Understanding these patterns will both advance resource management strategies for this ecologically vital group and elucidate EBLF responses to future climate change.

## 2. Results

### 2.1. Model Accuracy Evaluation

**Figure 1:**
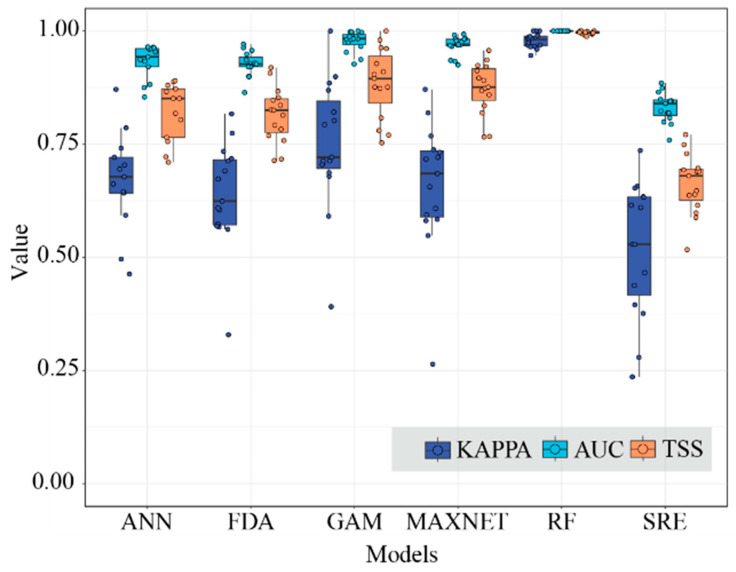
The Kappa, AUC, and TSS values of the six models.

In terms of simulation accuracy and reproducibility, RF and MAXENT demonstrated superior performance, with mean TSS and AUC values exceeding 0.8. In contrast, SRE exhibited relatively low mean TSS, AUC, and Kappa values during pre-evaluations for most species in subgenus *Camellia*, resulting in its limited participation in the construction of the ensemble model. The average TSS and AUC values generated by the remaining three models were within the range delimited by the extreme values exhibited by RF, MAXENT, and SRE; the average Kappa value exceeds 0.6. (Figure 1). Consequently, the final ensemble model predominantly integrated five models with optimal evaluation outcomes: ANN, FDA, GAM, MAXENT, and RF.

### 2.2. Climate Effects on Species Distribution

Correlation analysis identified seven key climatic variables influencing subgenus *Camellia* distribution: Bio2 (mean diurnal range), Bio3 (isothermality), Bio4 (temperature seasonality), Bio5 (max temperature of warmest month), Bio15 (precipitation seasonality), Bio18 (precipitation of warmest quarter), and Bio19 (precipitation of coldest quarter) (Table 1). Among these, Bio19 was the most important variable affecting the distribution of the subgenus *Camellia*, followed by Bio4. Species-specific responses varied: *C. brevistyla*, *C. japonica*, *C. sasanqua*, and *C. polyodonta* were primarily driven by Bio2 and Bio19; *C. grijsii*, *C. pitardii*, *C. saluenensis*, and *C. vietnamensis* by Bio4 and Bio18; *C. kissi*, *C. furfuracea*, and *C. edithae* by Bio4 and Bio19; *C. reticulata* by Bio3 and Bio4; *C. semiserrata* by Bio18 and Bio19; *C. chekiangoleosa* predominantly by Bio19; and *C. oleifera* by Bio4 and Bio2 (Table 1).

### 2.3. Current Distribution Patterns

**Figure 2:**
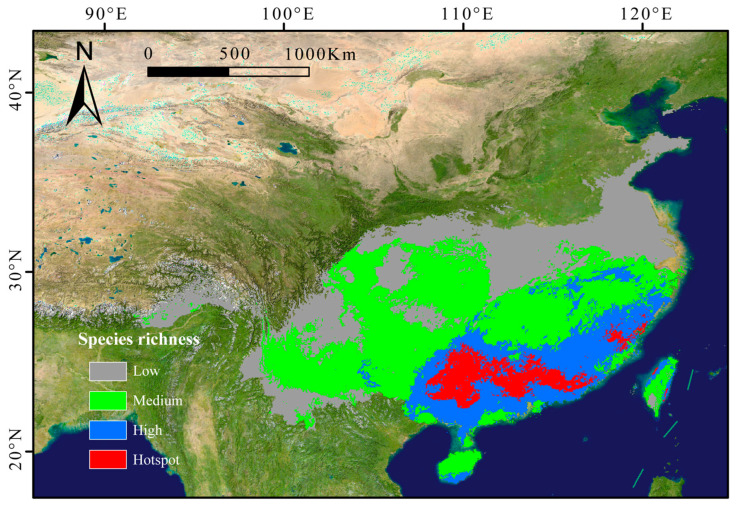
Predicted richness maps of subgenus *Camellia* in China in the current periods. Low richness: 1~3 species; medium richness: 3~6 species; high richness: 6~9 species; hotspot: > 9 species.

The subgenus *Camellia* was mainly distributed in southern China, encompassing regions such as Guangxi, Hunan, Sichuan, Guangdong, Fujian, Guizhou, Jiangxi, Hainan, and Taiwan. Richness hotspots clustered in the southern Nanling Mountains, eastern Guangxi Zhuang Autonomous Region, and Fuzhou (Fujian). A distinct latitudinal gradient was observed, with species diversity declining northward (Figure 2).

### 2.4. Effects of Climate Change on the Distribution Patterns of Subgenus Camellia

The model predicted that under future climatic scenarios (2081–2100), subgenus *Camellia* would maintain its core distribution in southern China while exhibiting differential range dynamics across emission pathways. The current total suitable area encompasses 2.25 × 10^6^ km^2^, comprising hotspot (1.4 × 10^5^ km^2^), high- (3.8 × 10^5^ km^2^), medium- (9.4 × 10^5^ km^2^), and low-suitability (7.9 × 10^5^ km^2^) zones (Table 2). Future climatic scenarios indicated an expansion trend in both the distribution range and proportion of suitable areas for the subgenus *Camellia* (Table 2).

**Figure 3:**
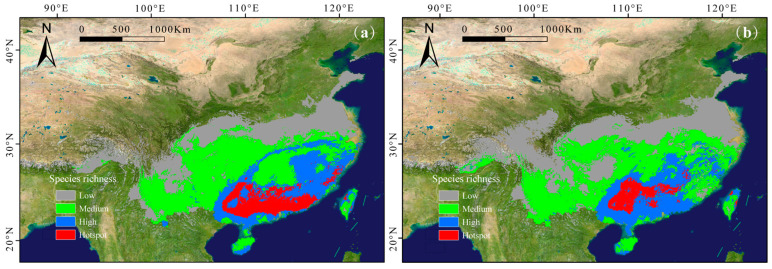
Predicted richness maps of subgenus Camellia under the two future emission scenarios, SSP126 (**a**) and SSP585 (**b**). Low richness: 1~3 species; medium richness: 3~6 species; high richness: 6~9 species; hotspot: > 9 species.

Under the SSP126 scenario, the richness hotspots and high-richness regions of subgenus *Camellia*, which were projected to be mainly concentrated in the eastern part of Guangxi Zhuang Autonomous Region, were projected to expand (Figure 3a). And the medium species richness and low species richness showed a decreasing trend (Table 2).

Under the SSP585 scenario, richness hotspots are predicted to concentrate in the Nanling Mountains and central and northeastern Guangxi Zhuang Autonomous Region (Figure 3b). The distribution patterns of subgenus *Camellia* are more sensitive to climate change, with the distribution hotspots and the medium species richness areas decreasing to varying degrees. The low species richness areas increased significantly, while high richness areas expanded slightly.

**Figure 4:**
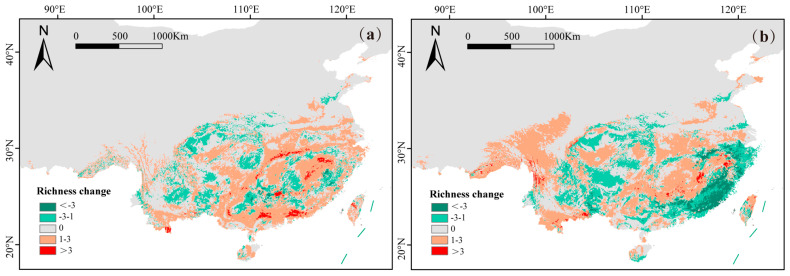
The species quantity change distribution of subgenus Camellia under future climatic scenarios, SSP126 (**a**) and SSP585 (**b**).

The species quantity changes in subgenus *Camellia* exhibited a distinct spatial pattern under different future climatic scenarios. Under the SSP585 scenario, significant species loss is predicted to primarily concentrate in currently high species richness areas, particularly in Fujian Province (Figure 4b). Species numbers in the Nanling Mountains are predicted to increase under the SSP126 climatic scenario (Figure 4a).

**Figure 5:**
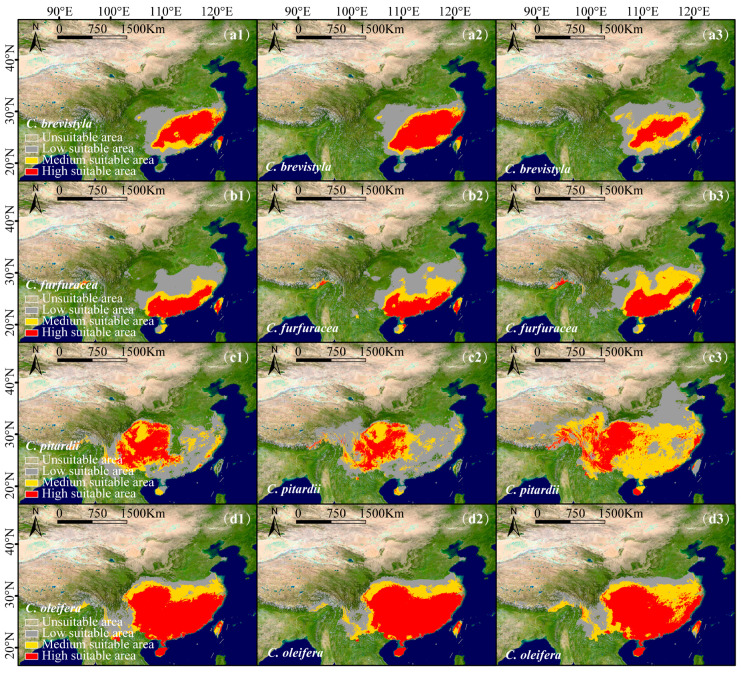
Predicted suitable areas of single species of the subgenus *Camellia* in China under current and future conditions: *C. brevistyla*: (**a1**–**a3**), *C. furfuracea*: (**b1**–**b3**), *C. pitardii*: (**c1**–**c3**), *C. oleifera*: (**d1**–**d3**). Panels 1–3 represent Current, SSP126, and SSP585 scenarios. The classification thresholds for each species are listed in Table S2.

From a single species perspective, under the SSP126 scenario, *C. brevistyla* (Figure 5(a1,a2)), *C. grijsii*, *C. kissi*, *C. furfuracea* (Figure 5(b1,b2)), *C. polyodonta*, *C. pitardii* (Figure 5(c1,c2)), *C. saluenensis*, *C. semiserrata*, *C. edithae*, *C. japonica*, *C. chekiangoleosa*, *C. sasanqua*, and *C. oleifera* (Figure 5(d1,d2)) exhibited expansions in total suitable areas (Table S1), while the highly suitable areas of *C. polyodonta*, *C. pitardii*, and *C. oleifera* are predicted to decrease. In contrast, while the suitable areas of *C. reticulata* and *C. vietnamensis* are predicted to contract, the highly suitable areas of *C. vietnamensis* are predicted to expand.

Under the SSP585 scenario, all species demonstrated significant range expansions (Figure 5(b3)) (Table S1). *C. pitardii* (Figure 5(c3)) had the most significant increase, with a total suitable area expanding by 1.38 × 10^6^ km^2^, while *C. polyodonta* displayed minimal growth, with only a 7.99 × 10^4^ km² increase. The highly suitable areas of *C. brevistyla* (Figure 5(a3)), *C. polyodonta*, *C. chekiangoleosa*, and *C. oleifera* (Figure 5(d3)) are predicted to decrease, while those of the other species are predicted to expand (Table S1).

### 2.5. Centroid Shift Under Climatic Scenarios

**Figure 6:**
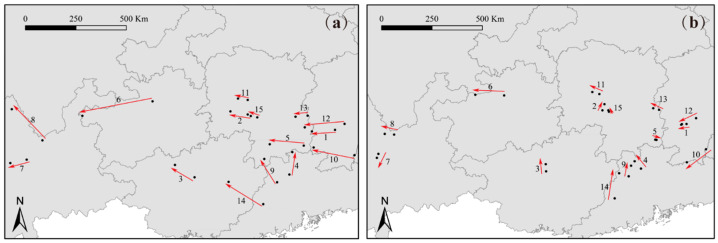
The director of centroid migration under the SSP126 climatic scenario (**a**) and SSP585 climatic scenario (**b**) for the 15 species of the subgenus *Camellia*.

The degree of centroid migration among subgenus *Camellia* species varied under different climatic scenarios (Table S3). Compared to the current period, under the SSP126 climatic scenario, *C. polyodonta* displayed the smallest centroid shift (5.01 km), with all centroids remaining within Chenzhou City, Hunan Province. In contrast, the centroid of *C. pitardii* exhibited the largest migration range, shifting 127.12 km westward from Zunyi City to Bijie City, Guizhou Province (Figure 6a). Under the SSP585 climatic scenario, *C. japonica* had the smallest change in distribution centroid, shifting westward from Xinhua County to Xupu County, Hunan Province, with a migration distance of 42.69 km. *C. pitardii* had the largest migration, with a migration distance of 318.15 km westward from Zunyi City to Bijie City in Guizhou Province (Figure 6b).

Overall, under future climate change, the distribution centroids of subgenus *Camellia* species are projected to shift slightly northwestward. The intensity of these shifts is notably greater under the SSP585 scenario compared to SSP126, reflecting the heightened impact of more extreme climatic conditions on species distribution dynamics.

## 3. Discussion

### 3.1. Overall Model Evaluation

Prior to constructing the final model, evaluating the performance of single models was essential to ensure accurate predictions. Different modeling algorithms have distinct advantages and limitations. For example, MAXENT is capable of fitting complex response curves even with limited occurrence data [26]. ANNs excel at processing complex, nonlinear relationships that are challenging to represent via traditional methods [27]. RF combines ensemble learning and parallel computing to achieve high computational efficiency and predictive accuracy [28]. The FDA provides robust discriminant analyses [29], whereas the SRE represents a traditional climate-envelope model with a relatively simple algorithmic structure [30]. Previous studies have demonstrated that reliance on a single modeling approach significantly limits predictive utility, as different models often yield divergent results even when applied to the same dataset [31,32]. To mitigate the biases associated with single-model approaches, the BIOMOD2 ensemble modeling platform integrates multiple models to simulate species distribution ranges, employing consensus methods, such as boundary values, commonality, or probability weighting, to generate robust predictions [33]. Biomod2 has been successfully applied to predict the distributions of various taxa, including *Haloxylon* [34], *Tsuga chinensis* [35], and *Ambrosia* L. [36].

The ensemble model leveraged the strengths of single models, achieving higher AUC, Kappa, and TSS values. This approach provides a more comprehensive and accurate simulation of the distribution of the subgenus *Camellia*, demonstrating superior predictive performance. Furthermore, the simulated distribution patterns closely aligned with the actual distribution of the subgenus *Camellia*, validating the scientific rigor and accuracy of the ensemble model.

### 3.2. Climatic Contributions to Distribution Patterns

The combined effects of temperature and precipitation play a critical role in shaping plant growth and distribution [37]. For example, temperature seasonality (Bio4), the maximum temperature of the warmest month (Bio5), and precipitation of the driest quarter (Bio17) were identified as key climatic factors determining the suitable habitat of *Phoebe bournei* [38]. Similarly, the distribution of *Manglietia fordiana* is constrained by precipitation of the driest month (Bio14), temperature seasonality (Bio4), precipitation of the warmest quarter (Bio18), and the minimum temperature of the coldest month (Bio6) [39]. For the subgenus *Camellia*, the most influential climatic factors are precipitation of the coldest season (Bio19) and temperature seasonality (Bio4). The distribution hotspots of the subgenus *Camellia* are predominantly located in southern subtropical China, where the warm, humid monsoon climate creates favorable conditions for species growth [40]. Extreme temperatures, whether excessively low or high, can adversely affect plant growth and development [41]. Temperature seasonality (Bio4), which reflects annual temperature variation, serves as an important indicator of seasonal temperature differentiation and climatic stability. According to the climate stability hypothesis, stable climatic conditions promote the availability of diverse natural resources, increasing species survival and adaptation [42]. In contrast, significant climatic fluctuations can disrupt plant growth rhythms and threaten species survival, leading to reduced species richness in regions with high temperature seasonality [43]. This pattern aligns with Rapoport’s rule, which posits that species richness decreases with increasing latitude due to greater temperature seasonality [44]. This trend explains the observed latitudinal gradient in species richness within the subgenus *Camellia*, with richness declining from south to north.

### 3.3. Species Distribution Dynamics

Paleoclimate analyses indicate that humid tropical and subtropical climates have been prevalent throughout China’s geological history, fostering the diversification of numerous plant groups adapted to warm, humid conditions [40]. The subgenus *Camellia*, a key component of EBLFs, is widely distributed across subtropical regions of China [7]. Currently, the subgenus *Camellia* exhibits high species richness in the southern Nanling Mountains, eastern Guangxi, and Fuzhou region of Fujian, with these areas supporting suitable habitats for more than nine species. Species richness within the subgenus *Camellia* decreases with increasing latitude, which is consistent with broader patterns of species richness in China, which generally increase from north to south, particularly in tropical and subtropical regions. For example, Cui et al. utilized microsatellite markers to demonstrate that genetic diversity in wild *Camellia oleifera* populations decreases with increasing latitude [45]. Similar latitudinal gradients have been observed in other plant families, including Magnoliaceae, which exhibit relatively high species richness in southeastern and southwestern China, gradually decreasing toward northern regions [46]. Fagaceae [47] and Lauraceae [48] also display comparable patterns, with species richness declining from south to north.

The southeastern region of China, characterized by a monsoon-influenced climate and diverse topography, supports a hot and humid environment that fosters high plant diversity and adaptability [49]. The Nanling Mountains, the largest east–west mountain range in southern China, serve as a natural barrier against cold air masses and are recognized as biodiversity hotspots [50]. This region functions as a Quaternary glacial refugium for *Camellia* species, preserving high levels of species and genetic diversity [21]. Fujian Province, located on China’s southeastern coast near the Tropic of Cancer, benefits from a warm, humid subtropical maritime monsoon climate, making it a core area for subtropical evergreen broad-leaved forests and an ideal habitat for the subgenus *Camellia* [51]. A previous study employing simple sequence repeat (SSR) marker technology similarly demonstrated that the subgenus *Camellia*, particularly *Camellia chekiangoleosa* populations, exhibits high genetic diversity in the Wuyi Mountains and Huaiyu Mountains [52]. Similarly, Guangxi’s subtropical humid climate and complex topography provide favorable conditions for the subgenus *Camellia*, which thrives in warm, acidic environments [40].

Under future climate scenarios, global warming and increased precipitation are projected to benefit the growth and distribution of the subgenus *Camellia* [53]. The models predicted the expansion of suitable habitats for the subgenus *Camellia*, with increases of 5 × 10^4^ km^2^ under the SSP126 scenario and 28 × 10^4^ km^2^ under the SSP585 scenario. However, despite the projected overall expansion of suitable habitats, areas of high species richness and distribution hotspots may initially increase before declining under more extreme climate scenarios. Under the SSP126 scenario, hotspots are expected to increase, benefiting from moderate warming and stable precipitation patterns. These balanced climatic conditions promote habitat expansion. Under the SSP585 scenario, hotspots exhibit significant reductions, primarily due to extreme warming and aberrant precipitation fluctuations. Such climatic conditions exceed the optimal thresholds for subgenus *Camellia* species [53], leading to intensified habitat fragmentation in existing hotspots (e.g., Fujian and Guangxi) and subsequent species loss. While some species may adapt to changing conditions through migration, this adaptation could result in declines in species richness and contraction of hotspot ranges in specific regions. Conversely, increased precipitation and humidity are expected to benefit most subgenus *Camellia* species, contributing to the overall expansion of suitable habitats [54]. Consistent with the general consensus that species’ suitable habitats will shift toward higher latitudes and altitudes under global warming [35], our findings indicate that the distribution center of the subgenus *Camellia* will shift primarily northwestward.

### 3.4. Resources Management and Study Limitation

Rich species diversity serves as a critical foundation for both ecological stability and genetic resource conservation [55]. It provides abundant genetic resources for the selection and domestication of superior varieties while ensuring ecosystem resilience. The establishment of germplasm repositories in ecologically suitable regions represents an effective strategy for biodiversity conservation and sustainable resource utilization. Our study demonstrates that the Nanling region constitutes a persistent species richness center for the *Camellia* subgenus. Notably, this tropical biodiversity hotspot encompasses multiple protected areas with established conservation infrastructure, including the Nanling National Nature Reserve, Chebaling National Nature Reserve in Guangdong, and Mangshan National Forest Park. Based on these findings, we propose the creation of a specialized *Camellia* germplasm repository in this region. Such a facility would systematically preserve both interspecific diversity across *Camellia* taxa and intraspecific genetic variation through provenance-based collections, thereby achieving integrated conservation of species diversity and population-level genetic heterogeneity.

This study also has some limitations. First, the spatial distribution of species occurrence data may reflect sampling-accessibility biases rather than true ecological patterns, as collection efforts were predominantly confined to anthropogenically accessible zones adjacent to transportation networks [56]. Such spatial sampling artifacts could propagate geographical biases into model predictions. Future investigations should implement unmanned aerial systems for systematic surveys of topographically challenging terrains to improve data representativeness. Second, while bioclimatic variables (temperature/precipitation parameters) formed the core of our environmental predictors, the model omitted other physiologically critical determinants, including edaphic properties (soil texture/pH), topographic complexity (slope/aspect/elevation), photoperiod variations, microbial symbioses, interspecific interactions, and anthropogenic pressures [57]. Furthermore, the current framework insufficiently addresses spatial constraints on dispersal capabilities and biogeographic barriers. Model refinement could incorporate high-resolution satellite-derived environmental layers, field-validated geospatial parameters, and mechanistic dispersal filters within ensemble forecasting algorithms to constrain ecological niche projections more realistically. Third, our approach adopted the conventional species-level niche conservatism assumption, disregarding potential local adaptations arising from population genetic divergence across isolation-by-distance gradients [58]. Subsequent research should integrate landscape genomic data to identify evolutionarily significant units, enabling the development of distinct ecological niche models for genetically clustered subpopulations. This hierarchical approach would substantially improve model biological realism by accounting for intraspecific niche variation.

## 4. Materials and Methods

### 4.1. Study Area

This study investigates the subtropical ecoregion of China, demarcated by the Qinling–Huaihe Line to the north and the Nanling Mountains to the south. The study area encompasses two principal geomorphological units: the middle-lower Yangtze River Basin and the Jiangnan Hills region. Three dominant topographic formations characterize this heterogeneous landscape: (1) undulating hill systems, most prominently represented by the Jiangnan Hills; (2) intermontane sedimentary basins, including the geologically significant Sichuan Basin; and (3) elevated karst plateaus, particularly the eastern Yunnan-Guizhou Plateau.

The heterogeneous topography supports diverse ecosystems ranging from riparian corridors and montane forests to karst wetlands. Climatically, the region exhibits distinct seasonal patterns under the influence of the East Asian monsoon system: humid summers and mild winters. These bioclimatic conditions sustain one of China’s most biodiverse ecoregions, harboring approximately 20,000 vascular plant species. The zonal vegetation comprises subtropical evergreen broadleaf forests dominated by Fagaceae, Lauraceae, Theaceae, and Magnoliaceae. However, this ecologically vital region faces increasing anthropogenic pressures, particularly from agricultural intensification, urban expansion, and resource extraction activities.

### 4.2. Occurrence Data Collection

The distribution data of the subgenus *Camellia* were systematically compiled from three principal sources: (1) the National Plant Specimen Resource Center (NPSRC; http://www.cvh.ac.cn/, accessed on 1 April 2025), (2) the Global Biodiversity Information Facility (GBIF; https://www.gbif.org/, accessed on 1 April 2025), and (3) the literature published between 2000 and 2024. Initial compilation yielded 1957 occurrence records encompassing 29 species.

To ensure data quality, records with incomplete or ambiguous locality descriptions were excluded. And for records lacking geographic coordinates but containing textual descriptions of collection sites, we georeferenced these locations using the Map Location geocoding service (https://maplocation.sjfkai.com/, accessed on 1 April 2025) with a positional accuracy threshold of 1 km. Then, spatial duplicates were removed using the “dismo” package in R v.4.1.3 software [59], retaining only one occurrence per species within each 0.1° × 0.1° grid cell.

Species with fewer than 25 records were excluded to minimize stochastic errors caused by narrow distribution areas or small sample sizes [60]. The final dataset comprised 15 species with 1455 validated distribution points (Table 3).

### 4.3. Environmental Variable Selection

The nineteen bioclimatic variables (2.5 arc-min resolution, version 2.1) were obtained from WorldClim [61,62], including current (1970–2000) and future (2081–2100) climate projections. Future climatic scenarios were derived from the CMCC-ESM2 model under two Shared Socioeconomic Pathways (SSPs): SSP126 (low emissions) and SSP585 (high emissions) [63]. The CMCC-ESM2 was selected for its advanced integration of Earth system components (atmosphere, ocean, land, biogeochemistry), alignment with IPCC AR6 standards, and capacity to simulate bioclimatic variables critical for plant distribution modeling [64]. As part of the CMIP6 ensemble, it ensures global assessment compatibility and effectively projects climate–vegetation interactions under SSP scenarios. Its proven accuracy in capturing East Asian regional dynamics (e.g., precipitation, temperature extremes) further supports habitat suitability predictions under future climate change [65]. The Shared Socioeconomic Pathways (SSPs) selected for this study represent an enhanced iteration of the Representative Concentration Pathways (RCPs), tailored to regional contexts. These pathways comprehensively incorporate socioeconomic dimensions, such as economic development, human development, technological advancement, demographics and human resources, lifestyle patterns, policy and institutional frameworks, as well as environmental and natural resource management [63]. This integrative approach effectively highlights the interplay between socioeconomic development trajectories and climate change risks.

**Figure 7:**
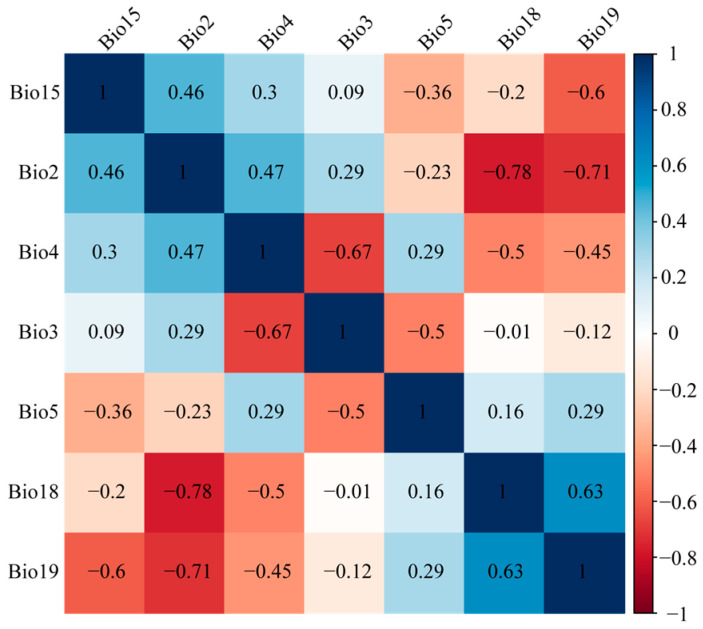
Correlations among the selected climatic variables.

High correlations among climate factors can lead to overfitting [66]. To eliminate highly correlated climatic variables and multicollinearity among variables, we adopted the following steps to filter the climatic variables ultimately used for simulation. First, we examined the pairwise correlations between variables and removed highly collinear variables. Then, we removed the less important climatic variable from each pair with the highest correlation (>|0.8|) using the “caret” package in R [67]. Finally, we selected seven variables that were inferred to make the highest contribution to the distribution of the subgenus *Camellia* for further analysis (Table 4, Figure 7).

### 4.4. Species Distribution Model Construction and Evaluation

The “Biomod2” ensemble modeling platform in R was used to simulate the suitable distribution range of subgenus *Camellia* for current and future periods. To overcome methodological constraints of single-algorithm approaches, we employed a consensus forecasting framework incorporating six complementary algorithms: Random Forest (RF), Maximum Entropy (MAXENT), Generalized Additive Models (GAMs), Flexible Discriminant Analysis (FDA), Artificial Neural Networks (ANNs), and Surface Range Envelopes (SREs). For each species, 1000 pseudo-absence points were randomly generated within the study extent. Data were partitioned into 75% training and 25% testing sets, with 10 replicates per model to quantify stochastic uncertainty. The single models with TSS (True Skill Statistics) values ≥ 0.7 were integrated using the weighted average method, and the ensemble model was constructed with TSS values as weights for simulation.

The model’s accuracy was assessed using the area under the receiver-operating characteristic curve (AUC), the true skill statistic (TSS), and Cohen’s Kappa coefficient (Kappa) [68,69,70]. TSS and Kappa are threshold-related metrics representing the net prediction success rate of the tested samples, with values ranging from −1 to 1; higher values indicate better model performs [71]. AUC is a threshold-independent evaluation method, with values ranging from 0 to 1; the values closer to 0 indicate poor predictive performance, while higher values reflect better simulation accuracy [72]. To identify the dominant climate variables influencing the suitable distribution of subgenus *Camellia*, we calculated the importance of each variable using weighted superposition based on their assigned weights in the ensemble model.

### 4.5. Species Distribution Patterns and Centroid Migration Analysis

The distribution patterns of 15 species within the subgenus *Camellia* were predicted using an ensemble modeling approach, followed by binarization based on threshold values. We first extracted the habitat suitability index corresponding to species distribution points and then calculated the mean and standard deviation. Thresholds were defined as the mean habitat suitability minus one standard deviation. Areas with index values above the threshold were deemed suitable distribution areas for the species, while those below were classified as unsuitable. We overlaid these binary distributions in ArcGIS v.10.8 to identify species richness patterns for the subgenus *Camellia* in current and future periods. Using the Reclassify function in ArcGIS toolbox, we categorized the species distribution into four levels based on intervals of three species: low (1–3 species), medium (3–6 species), high (6–9 species), and hotspot (more than 9 species). Higher category levels indicate areas suitable for a greater number of subgenus *Camellia* species. Then, we calculated the area of each richness category. Additionally, the habitat suitability for single species was categorized into four distinct tiers—unsuitable, low, medium, and high—using the natural breaks (Jenks) method.

The centroids of each species and the distances between these centroids at different times were calculated using the “raster” and “geosphere” packages in R [73,74]. The coordinates of the distribution points for each species were extracted, and the latitude and longitude means were the species distribution centroids. We calculated the distances between the current and future centroids of each species and analyzed the migration directions using ArcGIS.

## 5. Conclusions

Our study of species distributions highlights the importance of integrating multispecies data to predict the distribution patterns and conservation priorities of the subgenus *Camellia* under future climate change scenarios. Our findings indicate that the range sizes of most subgenus *Camellia* species are likely to expand and shift toward higher latitudes by 2070 in response to climate warming. The temperature seasonality and precipitation of the coldest quarter emerged as the dominant climatic variables influencing the distribution patterns of the subgenus *Camellia*. Although many subgenus *Camellia* species are projected to experience range expansions, the majority of their suitable habitats will remain vulnerable to increasing climate variability. Based on these findings, we propose the establishment of *Camellia* germplasm repository for the subgenus *Camellia* in its species richness hotspots, such as the southern Nanling Mountains. Additionally, areas experiencing declines in species richness should be prioritized for ecological restoration and habitat reconstruction to provide increased living space and resource availability.

## Figures and Tables

**Table 1:** The contribution of climatic variables on distribution ranges of 15 species in subgenus *Camellia* predicted by SDMs. Contribution rate in %.

Species	Bio15	Bio18	Bio19	Bio2	Bio3	Bio4	Bio5
*C. brevistyla*	9.80	9.22	36.11	16.90	7.74	14.43	5.80
*C. grijsii*	9.57	22.19	12.93	16.52	10.69	20.60	7.49
*C. kissi*	9.91	16.36	18.83	10.82	11.47	25.26	7.35
*C. furfuracea*	9.84	15.12	26.96	12.68	8.34	18.70	8.35
*C. polyodonta*	11.27	14.11	31.77	17.11	7.50	12.52	5.73
*C. pitardii*	7.63	23.34	12.05	13.25	8.28	26.71	8.74
*C. reticulata*	8.62	11.20	12.45	10.78	20.45	27.41	9.09
*C. saluenensis*	8.35	17.39	13.97	8.51	13.49	31.20	7.10
*C. semiserrata*	12.29	17.72	23.19	9.52	7.82	16.66	12.80
*C. edithae*	15.38	10.04	24.01	10.61	13.18	18.77	8.02
*C. japonica*	6.39	12.40	26.65	23.78	5.48	15.36	9.94
*C. chekiangoleosa*	17.17	12.02	35.85	10.96	6.05	10.72	7.23
*C. sasanqua*	10.47	10.70	22.37	23.11	9.31	14.43	9.61
*C. vietnamensis*	7.89	20.74	16.14	15.20	8.05	17.73	14.25
*C. oleifera*	8.98	16.98	15.28	17.93	5.93	24.81	10.09

**Table 2:** Species richness areas (km^2^) of subgenus Camellia in the current and future periods with different climatic scenarios.

Species Richness	Low (1–3)	Medium (3–6)	High (6–9)	Hotspot (>9)
Current	7.9 × 10^5^	9.4 × 10^5^	3.8 × 10^5^	1.4 × 10^5^
2081–2100 SSP126	7.5 × 10^5^	9.0 × 10^5^	4.4 × 10^5^	2.1 × 10^5^
2081–2100 SSP585	1.06 × 10^6^	9.3 × 10^5^	4.2 × 10^5^	1.2 × 10^5^

**Table 3:** The filtered occurrence data of 15 species of subgenus Camellia, utilized for subsequent analysis.

No.	Species	Number of Records
1	*Camellia brevistyla*	121
2	*Camellia grijsii*	27
3	*Camellia kissi*	47
4	*Camellia furfuracea*	49
5	*Camellia polyodonta*	27
6	*Camellia pitardii*	178
7	*Camellia reticulata*	79
8	*Camellia saluenensis*	74
9	*Camellia semiserrata*	29
10	*Camellia edithae*	26
11	*Camellia japonica*	147
12	*Camellia chekiangoleosa*	54
13	*Camellia sasanqua*	38
14	*Camellia vietnamensis*	27
15	*Camellia oleifera*	532
	Total	1455

**Table 4:** Climatic variables used in species distribution models.

Code	Environmental Variables	Unit
Bio2	Mean Diurnal Range	°C
Bio3	Isothermality (BIO2/BIO7) (×100)	-
Bio4	Temperature Seasonality	°C × 100
Bio5	Max Temperature of Warmest Month	°C
Bio15	Precipitation Seasonality	mm
Bio18	Precipitation of Warmest Quarter	mm
Bio19	Precipitation of Coldest Quarter	mm

## Data Availability

Data are contained within the article.

## Supplementary Materials

The following supporting information can be downloaded at https://www.mdpi.com/article/10.3390/plants14071137/s1: Table S1 Suitable area in 15 species of subgenus *Camellia* in the current and future different climatic scenarios and change trends compared to the current; Table S2 The classification thresholds for unsuitability, low suitability, medium suitability, and high suitability of 15 species; Table S3 Coordinates and distance variation in centroid migration in different climatic scenarios on the 15 species of subgenus *Camellia*.
